# A random Q-switched fiber laser

**DOI:** 10.1038/srep09338

**Published:** 2015-03-23

**Authors:** Yulong Tang, Jianqiu Xu

**Affiliations:** 1 Key Laboratory for Laser Plasmas (Ministry of Education) and Department of Physics and Astronomy, IFSA Collaborative Innovation Center, Shanghai Jiao Tong University, Shanghai 200240, China

## Abstract

Extensive studies have been performed on random lasers in which multiple-scattering feedback is used to generate coherent emission. Q-switching and mode-locking are well-known routes for achieving high peak power output in conventional lasers. However, in random lasers, the ubiquitous random cavities that are formed by multiple scattering inhibit energy storage, making Q-switching impossible. In this paper, widespread Rayleigh scattering arising from the intrinsic micro-scale refractive-index irregularities of fiber cores is used to form random cavities along the fiber. The Q-factor of the cavity is rapidly increased by stimulated Brillouin scattering just after the spontaneous emission is enhanced by random cavity resonances, resulting in random Q-switched pulses with high brightness and high peak power. This report is the first observation of high-brightness random Q-switched laser emission and is expected to stimulate new areas of scientific research and applications, including encryption, remote three-dimensional random imaging and the simulation of stellar lasing.

Disordered optics, which is the subject of light transport through non-uniform media, has attracted considerable interest and has numerous potential applications such as imaging, remote sensing, random lasers, and solar energy^1^. Among these areas, random lasers are especially important because of their unique properties and rich underlying laser physics, which are of fundamental scientific interest. Since the concept of the random laser was first introduced by Letokhov *et al.*^2^, random lasers have been realized using bulk powders, dye solutions containing particles, multilayered films, fiber configurations, non-uniform waveguides, and even atomic vapors^3^,^4^,^5^,^6^,^7^,^8^,^9^,^10^,^11^,^12^,^13^,^14^.

Unlike regular lasers, in which parallel mirrors are used to produce feedback and resonant modes, random lasers depend on multiple scattering in disordered media to trap light, where the interference of the scattered light results in resonant modes at particular frequencies^15^. Random lasers do not exhibit stationary resonance and thus display strong space- and time-dependent fluctuations in their emission properties, i.e., the emission direction, laser strength, and emission spectrum. In the development of random lasers, significant effort has been expended to improve the directionality of the laser emission^7^,^9^,^12^,^16^ and frequency selection^17^,^18^,^19^, and the use of fiber configurations has resulted in a new level of control^9^,^10^,^20^,^21^,^22^,^23^. However, efficient control of random laser emissions in the temporal regime has not yet been achieved, and the emission brightness of random lasers is comparatively low. Standard regular lasers, which have definite cavity modes, can be Q-switched with a conventional modulator to produce giant energy pulses and high peak power. Random lasers have a large number of randomly distributed modes that are strongly coupled together, which generate emissions in all directions at various positions and rapidly deplete the pump-accumulated energy. Consequently, energy cannot be effectively stored in random lasers, and their Q-values cannot be controlled using traditional techniques.

Here, we exploit strongly nonlinear effects in random fiber lasers to modulate the Q-value of the system and to achieve Q-switching. We use feedback from Rayleigh scattering to define the random cavity modes and stimulated Brillouin scattering (SBS) to improve the Q-value of the cavity quickly and significantly, thus efficiently combining random lasing and Q-switching in a simple fiber configuration. The random Q-switched fiber laser (RQFL) outputs stored energy over a short time interval, which greatly improves the emission brightness (peak power). Experiments have shown that the RQFL produces pulses with a peak power above 2 kW. Q-switched random lasers with such high peak powers can not only reveal rich underlying laser physics and related dynamics occurring in the interaction between light and disordered media but can also greatly enhance the applicability of random lasers. Immediate intriguing applications of high-power random lasers include speckle-free imaging^24^ and full-field optical coherence tomography^25^.

## Results

**Figure 1:**
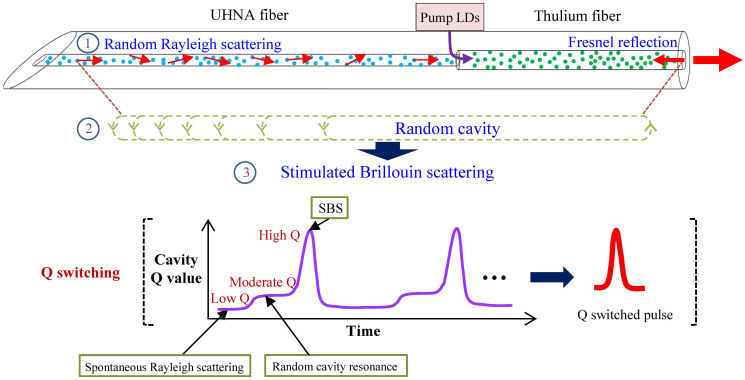
Apparatus and operating principle of a random Q-switched fiber laser. UHNA: ultra-high numerical aperture; LD: laser diode; SBS: stimulated Brillouin scattering; most of the power is outputted from the right-hand end of the fiber end.

Figure 1 displays a schematic of the apparatus and the operating principle of the RQFL. The laser system primarily consists of one piece of an ultra-high numerical aperture (UHNA) passive fiber that is fusion-spliced with one piece of a Tm^3+^ gain fiber. The suspended end of the UHNA fiber is cleaved at an angle of ≃10° to ensure that feedback is only produced by randomly distributed Rayleigh scattering. The open end of the Tm^3+^ fiber is perpendicularly cleaved to provide ≃4% Fresnel feedback and also acts as the output coupler. A high gain is achieved by using high-power laser diodes to pump the double-clad Tm^3+^-doped silica fiber. The unique properties of the passive fiber (small core area and large NA) enhance both the light intensity in the fiber core and the overlap integral between photons and phonons, facilitating light backscattering and reducing the SBS threshold.

As a proof of concept, the underlying mechanism of the random Q-switching process is described here (see Fig. 1). First, under pumping, spontaneous emission at ≃2-μm (near the gain peak) is generated in the Tm^3+^ fiber. During propagation of the spontaneous emission, micro-scale irregularities of the fiber core stimulate spontaneous Rayleigh scattering, especially in the UHNA fiber because of its specific fiber configuration. These randomly distributed Rayleigh scatterings act as a large number of frozen Rayleigh reflectors along the fiber^10^,^26^ and return a fraction of the spontaneous emission back to the laser cavity. The light signal that is enhanced solely by the Rayleigh-scattering feedback is still very weak, and the Q-value of the cavity remains at a low level. However, when these randomly distributed Rayleigh reflectors act synchronously with the ≃4% Fresnel reflection of the perpendicularly cleaved gain fiber facet, many closed feedback loops are activated, which results in the formation of a large number of random modes. These as-formed random cavity resonances increase the light intensity and consequently increase the Q-value of the cavity to a moderate level. Some random cavity modes are more highly overlapped with the gain peak of the Tm^3+^ fiber and are considerably amplified, which, in combination with the simultaneous linewidth narrowing due to Rayleigh scattering, finally stimulates the SBS processes. The occurrence of SBS dramatically increases the Q-value of the system to an extremely high level, and laser oscillations suddenly appear. Consequently, a giant pulse forms and is coupled out from the system. The export of the giant pulse depletes the intra-cavity stored energy, and stops the SBS process; thereby the Q-value of the cavity drops. Under continuous pumping, the gain and the Q-value of the cavity are recurrently driven up and down by the SBS process, thus generating random pulse trains.

**Figure 2:**
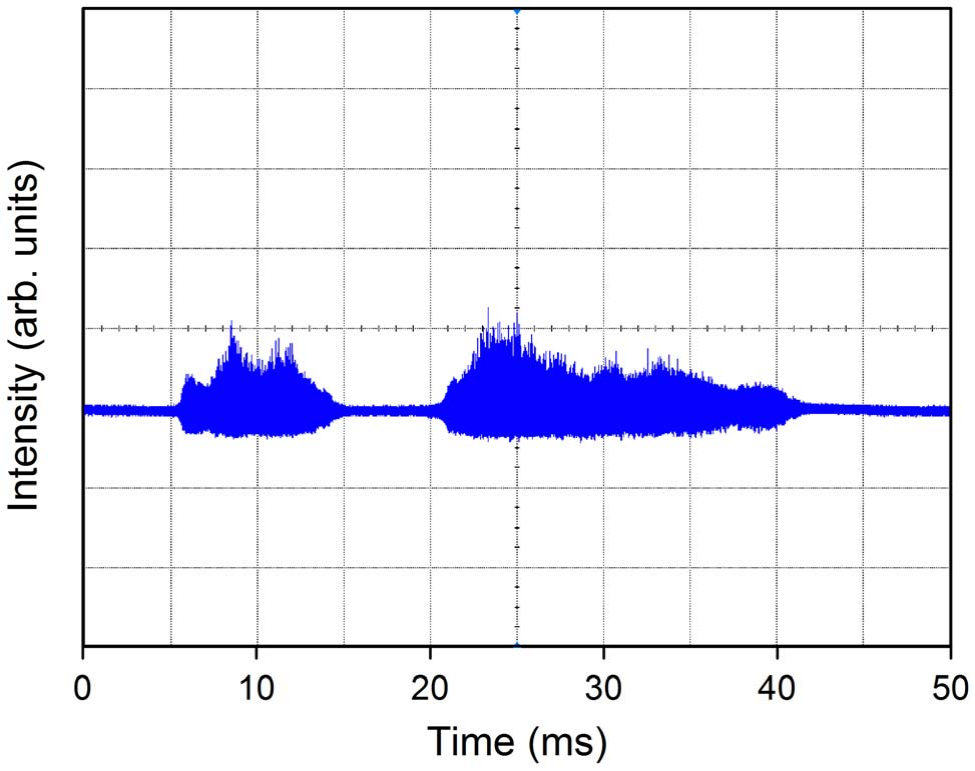
Random Q-switched laser pulses from the RQFL. The pump power is 4 W, and the average output power fluctuates from ≃10 mW to ≃50 mW.

For low pump power, no pulse emissions are observed because spontaneous Rayleigh scattering and random cavity resonance cannot increase the Q-value of the system to a sufficiently high level. Thus, the entire cavity remains in a high loss state. However, an entirely different scenario results when the pump power is increased to a certain level (≃4 W). High-intensity pulses are occasionally observed (see Fig. 2). These giant pulses appear occasionally and vanish suddenly, but this random pulsing is always observed provided the pumping is sustained. At this point, the random Q-switching regime arises. Each generated single pulse has an extremely high intensity (with a peak power above 1 kW). This generation of giant pulses in the random Q-switching state results from rapid enhancement of the Q-value of the cavity by the stimulated SBS process. Only those modes that have a sufficiently high intensity to stimulate SBS can overcome the total high system loss, thus improving the Q-value of the cavity rapidly and significantly. In this domain, the output emission exhibits significant fluctuations in both the temporal and spectral regimes (which vary significantly among measurements). This behavior occurs because of the strong coupling among a large number of spatially overlapping random modes; competition among these modes results in a highly chaotic emission^27^,^28^. The random resonance can occasionally cause the Q-value of the system to reach a high level (leading to giant pulse generation); however, at other times, the Q-value cannot be increased to such a level, and no giant pulses are produced.

**Figure 3:**
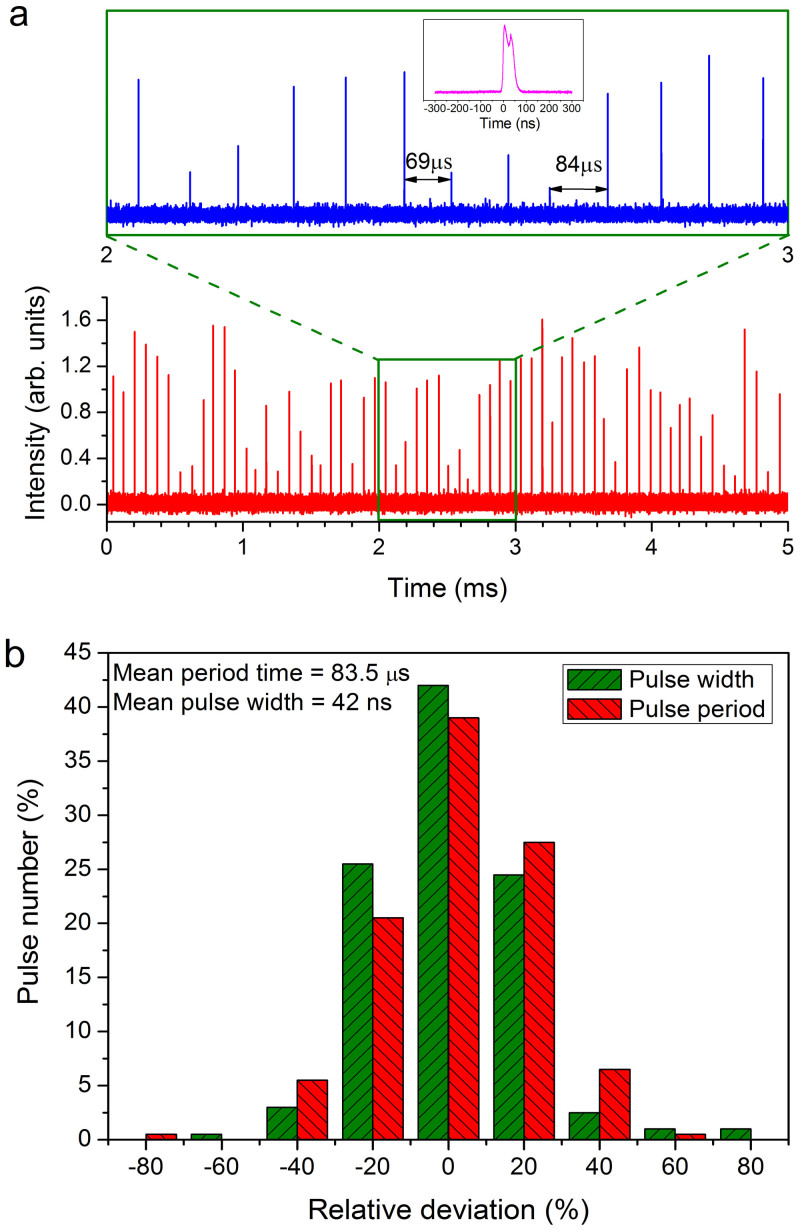
Random Q-switched laser pulse train of the RQFL measured at a pump level of 8 W (≃1 W average output). (a), Pulse train with different time scales; the magnified pulse train (upper panel) clearly exhibits an uneven pulsing period; the inset shows a typical single pulse; (b), statistical properties of the pulse train: the horizontal axis shows the deviation relative to the average value, where every 20% variation relative to the average value is denoted as one range (e.g., 0% denotes deviations between −10% and 10%, and 20% denotes deviations between 10% and 30%); the vertical axis shows the pulse number percentage with respect to the total pulse number; a total of 200 consecutive pulses is used for statistical analysis of the pulse train.

As the pump power increases, the pulse intensity increases, and the random giant pulses become denser (the number of pulses for a fixed time interval increases). The concentration of random pulses as a function of the pumping level can be found in the supplementary information (Fig. S1). To elucidate the random pulsing characteristics of the RQFL, another pulse train is measured at a comparatively higher pump level (8 W) over a smaller time window (Fig. 3a). The results show large fluctuations in both the peak intensity and the pulsing period. To investigate the random pulsing characteristics in detail, we sample 200 consecutive single pulses to statistically analyze the variation in the pulsing period and the pulse width: the results are shown in Fig. 3b. Here, the pulse width is the envelope value of each pulse, and the deviations in the pulsing period and the pulse width are given relative to their respective mean values. Greater than 50% of the pulses show >20% deviation in both the pulsing period and the pulse width, and some pulses even demonstrate deviations in the period and the pulse width above 60%. The standard deviation of the period with respect to the corresponding mean value (i.e., the ratio of the standard deviation to the mean value) is ≃10%. The standard deviation of the pulse width with respect to the mean value is ≃17%. The large fluctuations in the pulsing characteristics (period, pulse width and intensity) clearly show that the laser emission originates from random cavities (the cavity length is random and fluctuates with time) and also demonstrates the unique features of random Q-switched lasers.

The shape and width of a single pulse are unpredictable. The pulse shape differs significantly among pulses (see Fig. S3 in the supplementary information), and the pulse envelope width fluctuates from almost 20 ns to over 70 ns (see Fig. 3b). These pulses primarily consist of two sub-pulses (see the inset of Fig. 3a), each with a pulse width of ≃20 ns. The sub-pulse width is consistent with the phonon decay time of ≃20 ns in silica fiber^29^. This result confirms that the significant improvement in the Q-value of the cavity results from the SBS process aided by acoustic waves.

**Figure 4:**
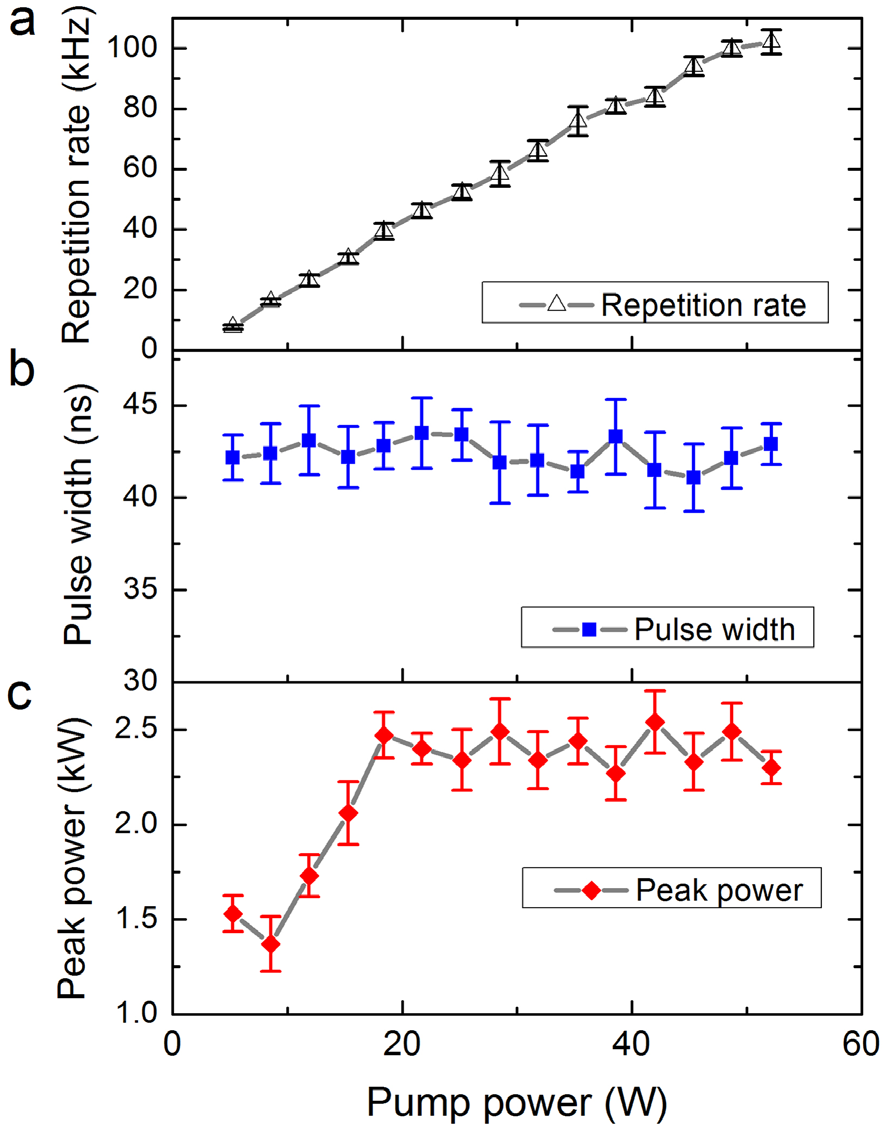
Pulsing characteristics of the random Q-switched fiber laser. (a), repetition rate at different pump power levels; (b), pulse width at different pump power levels; (c), pulse peak power at different power levels; each pulse width and repetition rate measurement corresponds to 100 consecutive pulses, and the final values correspond to ensemble averages of 10 measurements; the peak power is obtained by dividing the 10-time ensemble-averaged output power by the average pulse width; the solid lines are provided to guide the eye; the error bars indicate the standard deviations.

As the pump power is increased, the output power increases accordingly, and the pulse density continues to increase. The random pulsing regime can be sustained up to the maximum available pump power (≃50 W). The evolution of several pulsing characteristics (repetition rate, pulse width and peak power) of the RQFL as a function of pump power is shown in Fig. 4. The linear increase of the repetition rate with pump power is similar to that of conventional passively Q-switched lasers^30^, which clearly demonstrates the Q-switching feature of our random fiber laser. The pulse envelope width fluctuates around a horizontal line (≃42 ns) but does not significantly shrink or spread as the pumping strength increases (Fig. 4b). This behavior is very different from that of conventional Q-switched lasers, for which the pulse duration depends strongly on the pump power^30^ and is sensitive to the turn-on time of active Q-switches or the recovery time of passive Q-switches. This behavior arises because the Q-switched pulses in our fiber laser are stimulated by the SBS process which has a constant relaxation time (the phonon lifetime) for a given host material. Another key feature of our RQFL is that it operates in the high peak power regime. The maximum peak power is above 2 kW (Fig. 4c), which represents the first evidence of high peak power performance in random lasers. The peak power is clamped at approximately 2.3 kW under high pump levels, demonstrating the quantization-like behavior of the pulse energy in the RQFL. These high peak power random Q-switched lasers can considerably broaden the applicability of random lasers in areas requiring high-power emissions.

**Figure 5:**
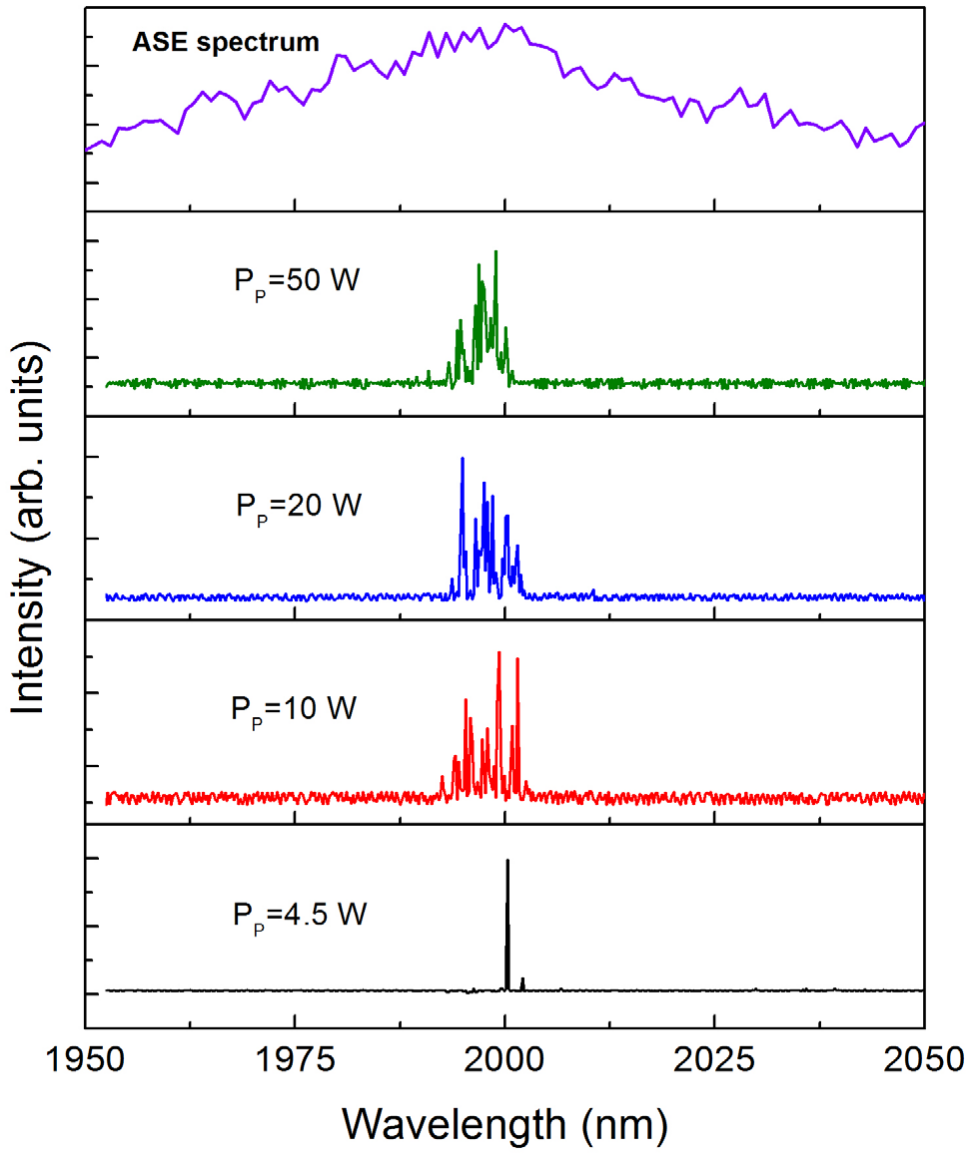
Spectral properties of a random Q-switched fiber laser. The top panel shows the spontaneous emission spectrum of the gain fiber. The spectra are measured at different pump power levels, and all of the spectra are shown on a linear scale.

Some of the characteristics of the RQFL can be observed from the measured laser spectra (Fig. 5). At low pump power levels, few modes can go into oscillation because of the limited gain, and the spectrum varies significantly among measurements. Details of the variation in the spectral features at a fixed pump power can be found in the supplementary information (Fig. S4). At higher pump power levels, more spectral peaks appear because a greater number of random modes obtain a sufficient gain to overcome the cavity loss. Further increasing the pump power level lifts up the spectral envelope, causing more random modes to converge together. The overlap of multiple peaks in the spectral envelope clearly demonstrates the characteristics of coherent random lasers^20^. The central spectral wavelength of this RQFL is in good agreement with the gain peak of the fiber and shows a negligible dependence on the pump power level, which is also consistent with the behavior of conventional random solid-state lasers^18^. In random lasers, a large number of random modes compete with each other for gain and cancel each other out in the cavity. The modes with frequencies near the peak gain are more likely to increase their gain over those modes that are far from the peak gain and are thus able to survive.

## Discussion

In conventional random lasers^3^,^4^,^5^, a large number of random cavity modes exist simultaneously across the disordered material (as both localized and extended modes), simultaneously overlapping and competing with each other. Therefore, the random laser emission exhibits a strong spectral dependence and an angular dependence (i.e., the laser emission radiates in all directions) and dissipates energy at all points in space and time. This behavior renders energy storage impossible in random lasers. However, conventional Q-switching techniques (e.g., in acousto-optic and electro-optic modulators) are based on periodic energy storage and depletion and therefore cannot be transplanted directly into random systems to achieve high-energy pulsing operation.

In contrast, in our system, we first adopt a fiber configuration to confine the random emission in one dimension and then use a UHNA fiber to strengthen the Rayleigh scattering and realize strong random cavity resonance (i.e., to form random cavity modes). In addition, we adopt LD-pumped gain fibers to achieve a high gain (orders of magnitude above that of conventional random lasers) and store energy. Finally, we use the SBS process to dissipate the accumulated energy. The interplay between the pumping-induced energy storage and the depletion of energy in the SBS process acts as an effective Q-switch and produces a recurrent modulation of the Q-value of the system.

Our RQFL originates from random cavity modes and exhibits chaotic behavior (similar to that produced in conventional random lasers); however, the Q-switched state and high brightness of the RQFL make it completely different from conventional random lasers. Our laser system operates in a random Q-switched regime (in which giant pulses can be produced), whereas the emission from conventional random lasers is either completely stochastic^27^ or stationary CW output^10^. The pulsing state of our RQFL is very similar to that of traditional Q-switching operation but exhibits high randomness in the pulsing intensity, pulsing period and pulse shape because the RQFL originates from random cavity resonance. This random cavity resonance is caused by randomly distributed Rayleigh scattering and exhibits a random cavity length. However, the high Q-value of the cavity is maintained by the SBS process, such that the giant pulse width is commensurate with the SBS relaxation time (approximately tens of nanoseconds).

## Conclusion

We used random resonance induced by Rayleigh scattering and a nonlinear optical process (SBS) in high-gain fibers to investigate the Q-switching characteristics of random lasers in one dimension for the first time. Modulation of the Q-value in the cavity results in recurrent storage and extraction of the random cavity energy (which manifests as a pulsing regime), thereby realizing a random emission with high brightness. Our high-brightness random Q-switched laser can be extremely useful in application areas that require light sources with low coherence and high intensity, such as imaging^24^, full-field optical coherence tomography^25^, and focusing through random media^31^. The random Q-switched laser concept demonstrated herein can be straightforwardly extended to other wavelength regimes (e.g., visible, mid IR, and far IR) and other random laser configurations (e.g., bulk powders, multilayer semiconductor films, and scatterers in dye solutions). Instead of the SBS effect, other nonlinear optical effects (such as Raman scattering and four-wave mixing) may also be used to switch random lasers. The transition of random lasers from CW or completely chaotic regimes to Q-switched states offers various unique advantages that should open up new avenues for random lasers, both for potential applications and for fundamental scientific research combining laser physics, nonlinear optics and fiber optics with random scattering theory (new lasing theory in which Q-switching/mode locking is mixed with random scattering).

## Methods

A double-cladding pumping technique is used to achieve a high gain. The laser gain medium is a double-clad Tm^3+^-doped silica fiber (10/130 μm, 0.15/0.46 numerical aperture (NA)) with a Tm^3+^ doping concentration of ≃2 wt.% and a cladding absorption of ≃3 dB/m (at 793 nm). The pump sources are two 35-W 793-nm laser diodes (LDs) with an output fiber pigtail of 100/125 μm. The pump light is launched into the gain fiber through a (2 + 1) × 1 fiber combiner with a coupling efficiency of ≃95%. The fiber combiner has a signal fiber of 10/125 μm (NA of 0.15/0.46), which is almost perfectly matched to the gain fiber. The pump fiber of the combiner has the same parameters as the pigtail fiber of the pump LDs. The backscattering fiber is a UHNA passive fiber (with a total length of 50 m in this experiment) with a core diameter of 3.5 μm (NA of 0.41) and a clad diameter of 125 μm.

One end of the UHNA fiber is fusion-spliced to the signal fiber of the combiner, and the other end is cleaved at an angle of ≃10° to eliminate parasitic reflections and to ensure that the feedback from this fiber end only results from randomly distributed scattering (Rayleigh scattering). One end of the Tm^3+^ fiber is fusion-spliced to the output signal fiber of the combiner, and the other end is perpendicularly cleaved to provide ≃4% Fresnel feedback for the laser radiation. Propagation testing from a low-power 2-μm source shows that the total splice loss, which includes the loss for the UHNA fiber, the signal fiber of the combiner and the Tm gain fiber, is approximately 1.5 dB (primarily from the fusion point between the UHNA fiber and the combiner signal fiber).

A 3.2-m-long Tm^3+^ fiber is wrapped on a convectively cooled copper drum with a diameter of 10 cm. The laser power is outputted from the right side of the Tm^3+^ fiber. At the output end, a dichroic mirror (R > 99.9%@793 nm, 0°) is used to filter the residual pump light. The laser output power is measured with a power meter (FieldMax II-top, Coherent Co.), and the laser spectrum is recorded with a triple-grating spectrometer (Zolix Co.) with a spectral resolution of 0.2 nm. The laser pulsing dynamics are measured with a 2-GHz Agilent oscilloscope combined with a 1-GHz InGaAs detector.

## Supplementary Material

Supplementary Information supplementary information
