# Supplementary material for: A random Q-switched fiber laser
Source: Sci Rep. 2015 Mar 23;5:9338. doi: 10.1038/srep09338 (PMC4369747; doi:10.1038/srep09338)
Supplement: Supplementary Information [file srep09338-s1.docx]

**A random Q-switched fiber laser**

Yulong Tang and Jianqiu Xu^*^

Key Laboratory for Laser Plasmas (Ministry of Education) and Department of Physics and Astronomy, Shanghai Jiao Tong University, Shanghai 200240, China

*Correspondence should be sent to Y. T.: [yulong@sjtu.edu.cn](mailto:yulong@sjtu.edu.cn)

1. **Laser pulse trains at different power levels**

With pump power over ~4.4 W, more consecutive pulse train can be observed on the oscilloscope in certain time scales. Under two different time scales, we measured the pulse train of the random Q switched fiber laser (RQFL). In the first time scale, we sampled the pulse in a 10 ms time window at several pump power levels, and the results are shown in Fig. S1. At different pump power levels, the laser pulse train shows similar randomness but the pulse period (pulsing repetition rate) decreases with pump power. This decrease of the pulsing period clearly demonstrates the concentration of pulsing in RQFLs.


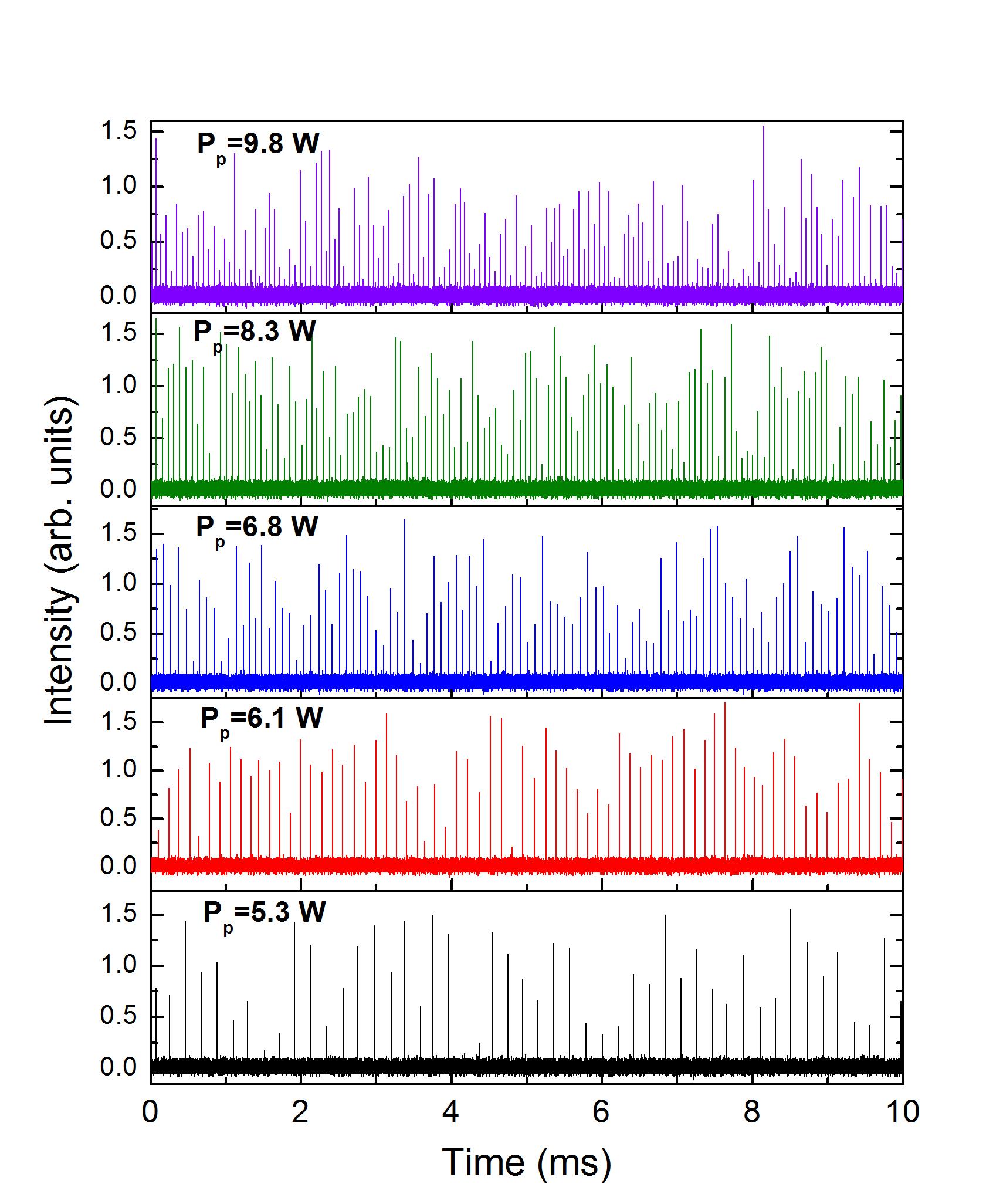


Figure S1. Laser pulse trains of the RQFL measured at several low pump levels in a 10-ms time window.

At all power levels, the random Q switched laser operates in the random pulsing regime, instead of CW mode. The random Q switching state can be sustained up to the highest pump power (~70 W). At high power levels, more pulses are observed over a fixed time interval. Therefore we also measured the pulse train in a smaller time window (200 μs) to show the characteristics of pulse trains, and the results are shown in Fig. S2.


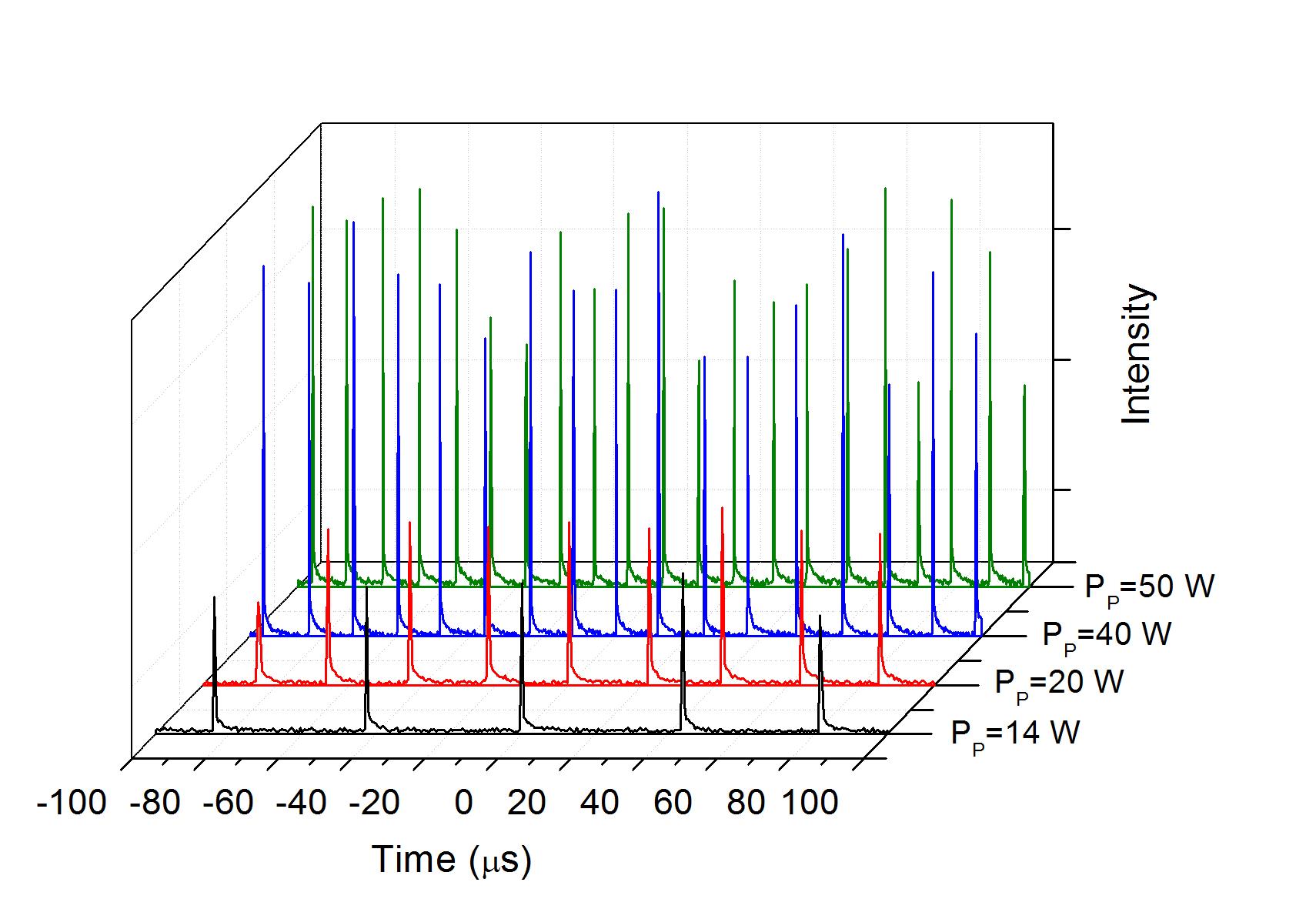


Figure S2. Laser pulse trains of the RQFL at several high pump levels measured in a comparatively shorter time window (200 μs).

1. **Single pulses measured at different times**

In this random Q switched fiber laser, the laser pulse shows great fluctuations in both pulse shape and pulse envelope width. To make this clear, we measured single output laser pulses at different times but under the same pump level. The results are shown in Fig. S3. Variation of the pulse width and pulse shaped can be clearly observed. Under other pump power levels, the measured single pulse shows similar changes with time.


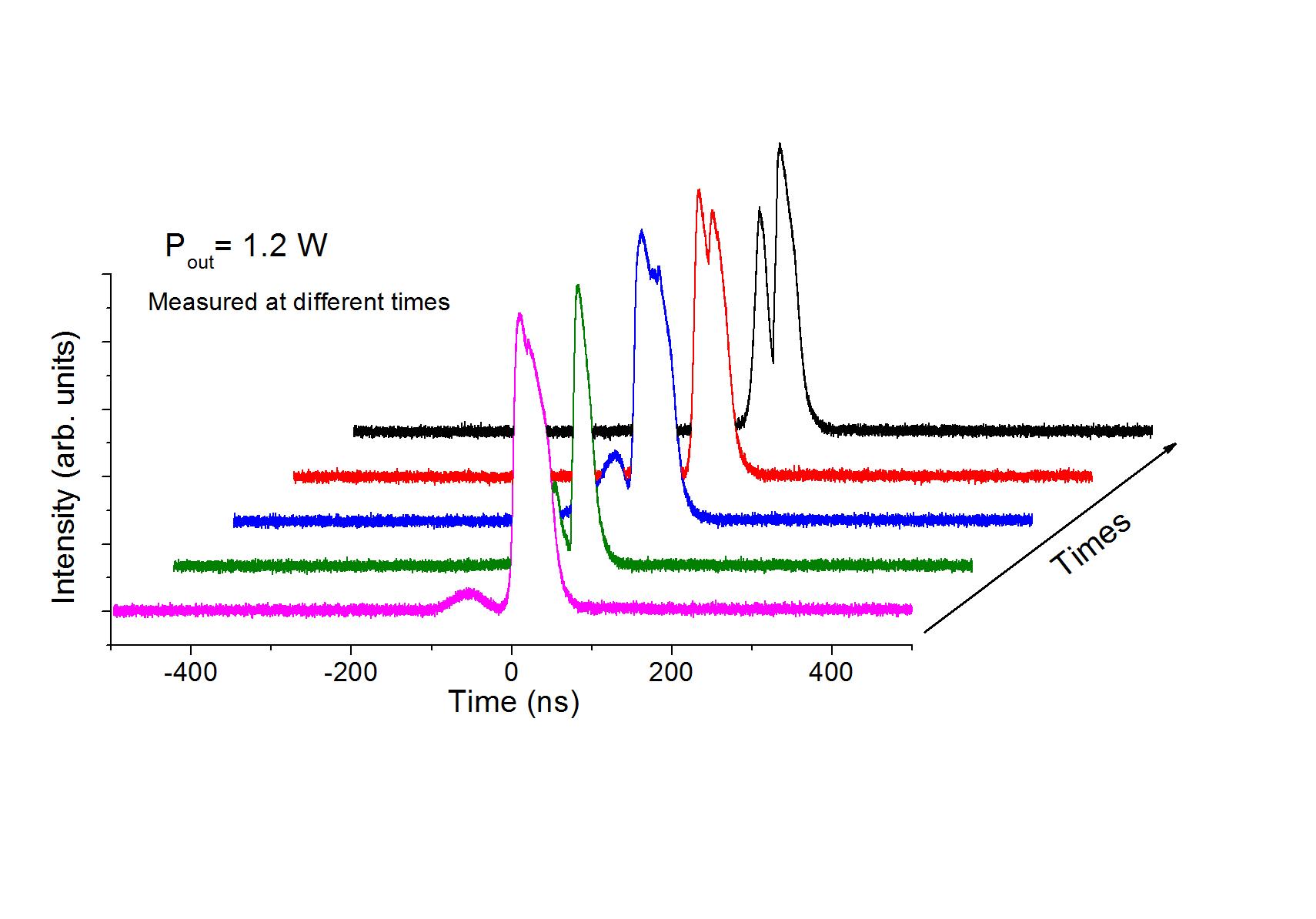


Figure S3. Single laser pulses of the RQFL measured at different times and under the same pump power of 9.8 W (output power of 1.2 W).

1. **Spectra measured at different times**

The emission spectra of the RQFL measured under the same pump level and at different times are shown in Fig. S4. Under the same pump strength, the emission spectrum is completely unpredictable due to its origination from random cavity modes and its evolution correlated with nonlinear scattering processes. This strong time-dependent spectrum feature clearly indicates the randomness of the mode structure of this Q switched random fiber laser (a large number of overlapping modes exist simultaneously). The spectrum shape and width (also the mode number) change significantly among measurements, although the envelope peak maintains around 2 μm. Under other pump levels, fluctuations of the spectrum with time show a similar behavior.


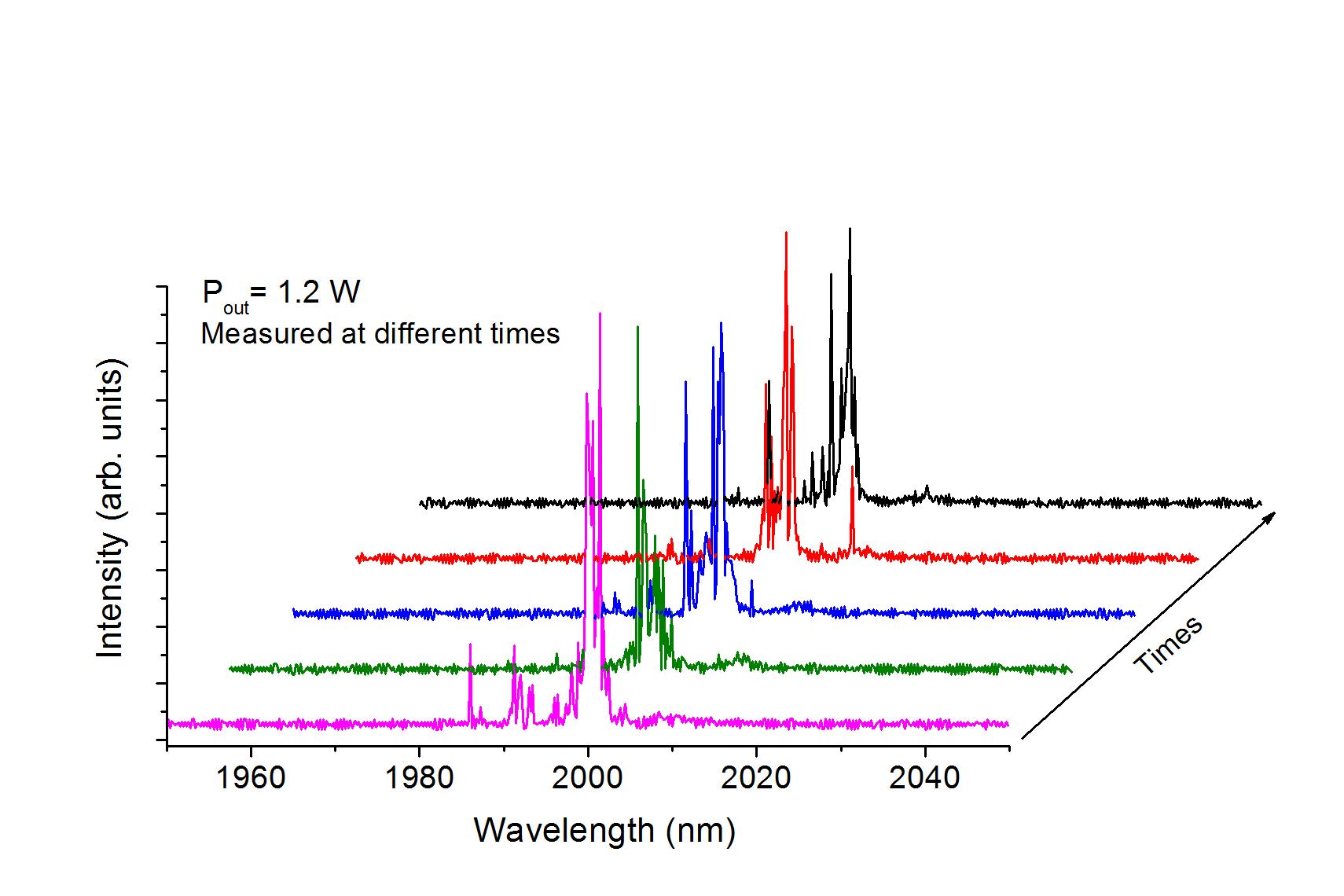


Figure S4. Spectra of the RQFL measured at different times and under the same pump level of 9.8 W (1.2 W output power).
